# Comparation of the effectiveness of conventional needle irrigation and photon-induced photoacoustic streaming with sodium hypochorite in the treatment of teeth with apical periodontitis: a randomized clinical trial

**DOI:** 10.1186/s12903-024-04055-6

**Published:** 2024-03-02

**Authors:** Jian Zhao, Shengxuan Wu, Yuting Huang, Yuxuan Zhao, He Liu, Qianzhou Jiang, Ya Shen, Minle Chen

**Affiliations:** 1 Department of Endodontics, Guangdong Engineering Research Center of Oral Restoration and Reconstruction, Guangzhou Key Laboratory of Basic and Applied Research of Oral Regenerative Medical, Affiliated Stomatology Hospital of Guangzhou Medical University, Guangzhou, 510182 Guangdong China; 2 Department of Stomatology, The Third Affiliated Hospital of Guangzhou Medical University, Guangzhou, 510150 Guangdong China; 3 Division of Endodontics, Faculty of Dentistry, University of British Columbia, Vancouver, BC V6T 1Z3 Canada

**Keywords:** Apical periodontitis, Laser therapy, Photon-induced photoacoustic streaming, Root canal irrigants, Root canal Therapy

## Abstract

Photon-initiated photoacoustic streaming (PIPS) with an Er: YAG laser has been introduced in root canal treatment to improve irrigation and facilitate the removal of bacteria in the root canal system. This study aimed to compare the antibacterial effectiveness of two different root canal irrigation techniques, conventional needle irrigation (CNI) and PIPS, using 1% sodium hypochlorite (NaOCl), in the treatment of teeth with apical periodontitis. Sixty patients with a total of sixty teeth affected by apical periodontitis were included in this study. The teeth underwent root canal therapy, and after mechanical instrumentation, they were randomly assigned to two groups (*n* = 30) based on the final irrigation protocol: CNI or PIPS with 1% NaOCl. Bacterial suspensions in the root canals were evaluated using Adenosine 5’-triphosphate (ATP) assay kit after mechanical instrumentation and after final irrigation. Then, a follow-up was conducted after 7 days. The results revealed that final irrigation significantly reduced ATP values in both the CNI and PIPS groups (*P* < 0.001). The ATP values after final irrigation was greater in the CNI group compared to the PIPS group (*P* < 0.001). After a 7-day follow-up, percussion tenderness and fistula were significantly resolved in both groups (*P* < 0.05). A multivariate linear regression model was used to identify the factors that influence post irrigation ATP values. The analysis demonstrated that pre-operative percussion tenderness (*P* = 0.006), the presence of a fistula (*P* < 0.001) and the method used in the final irrigation (*P* < 0.001) had a significant impact on the ATP value after final irrigation. These results indicate that employing PIPS with 1% NaOCl as the final irrigation protocol exhibited superior antibacterial effectiveness and has the potential to enhance clinical outcomes in the treatment of teeth afflicted with apical periodontitis.

## Introduction

Apical periodontitis primarily occurs due to a bacterial infection characterized by highly structured and resilient mature oral biofilms [1, 2]. These biofilms consist of bacterial colonies embedded in a highly hydrated extracellular polymeric substances (EPS) matrix, making them challenging to disinfect effectively [1, 2]. Modern endodontic treatment procedures aim to eliminate microorganisms during root canal preparation and disinfection. However, the complex anatomy of the root canal system can harbor microorganisms, making effective decontamination challenging. Studies have shown that the chances of complete healing in teeth affected by apical periodontitis are 10–15% lower compared to unaffected teeth [3]. Therefore, additional root canal irrigation strategies have been recommended to supplement endodontic disinfection procedures and address the limitations of instrumentation [4, 5].

An ideal irrigation protocol should not only efficiently disinfect the root canal system chemically but also physically disinfect by removing the biofilm and planktonic bacteria through shear stresses exerted on the root canal wall [6]. Laser-assisted irrigation has been proposed as an adjunct in endodontic treatments, offering a suitable method for removing the smear layer and achieving deep disinfection of the root canal system [7, 8]. Photon-induced photoacoustic streaming (PIPS) with a low-pulse-energy Er:YAG laser (20 mJ) and short pulse duration (50 µs) has been introduced in root canal treatment to aid in the removal of bacteria from the root canal system [9]. This technology relies on the mechanism of bubble cavitation, involving the generation of strong shockwaves due to the collapse of bubbles in a fluid, and has shown effectiveness in removing the smear layer from surrounding walls [10, 11]. It has been demonstrated that PIPS activation resulted in fewer apical bacteria/biofilm compared to ultrasonic activation [12]. Furthermore, our preliminary in vitro research has confirmed the strong bactericidal effect of PIPS combined with sodium hypochlorite (NaOCl) in the apical region of the root canal system [13]. Considering the current circumstances, it is plausible to incorporate PIPS-assisted chemical disinfection alongside the conventional mechanical preparation to enhance sterilization in the apical region and promote lesion healing. However, clinical research on endodontic treatments to date has shown promise but has been limited [14–16].

The objective of this study was to compare the antibacterial effectiveness of two different root canal irrigation techniques, conventional needle irrigation (CNI) and PIPS, using 1% NaOCl, in the treatment of teeth with apical periodontitis. The hypothesis tested was that PIPS with 1% NaOCl as a final irrigation protocol would demonstrate equivalent effectiveness, with no significant difference, compared to CNI. This assessment encompasses antibacterial activity and improved clinical symptoms, including reduced percussion tenderness and the resolution of fistula, following primary root canal treatment for apical periodontitis.

## Materials and methods

### Ethics approval

Ethics approval and Study Registration Approval for the investigation was obtained from the Ethics Committee of Stomatology Affiliated Stomatology Hospital of Guangzhou Medical University (No. XJS202001). The study was registered at the Chinese Clinical Trial Registry (ID: ChiCTR2000037446). Informed consent was obtained from all the participants and/or their legal guardians. All procedures performed involving human participants were in accordance with the ethical standards of the ethics committees and with the 1964 Helsinki declaration and its later amendments or comparable ethical standards.

### Sample size calculation

The required sample size was calculated using G power 3 software (Franz Faul, University of Kiel, Germany) to facilitate comparison of 2 experimental groups with significance level of 5%, statistical power of 80%, equivalence limit of 15%, and effect size of 0.58, which was based on a previous study [17]. A sample size of 30 teeth per group was determined.

### Eligibility criteria

Patients scheduled to undergo root canal treatment were selected for the study. Each patient received a full explanation of the treatment procedures, as well as the associated potential benefits and discomforts.

Inclusion criteria: Healthy adults (American Society of Anesthesiologists I, ASA I) between 18 and 65 years of age, with teeth having completely formed root apices, necrotic pulps, and radiographically verified apical periodontitis (minimum size ≥ 1.0 × 1.0 mm), and an apical alveolar bone resorption area with a diameter ≤ 5 mm on X-ray films [18]. Informed consent was obtained from all individual participants included in the study. Patients with good oral hygiene were included.

Exclusion criteria: Patients with a history of previous pulpotomy, pulpectomy, or root canal treatment; immunocompromised patients (e.g., diabetes mellitus, AIDS, hepatitis B, hepatitis C, tuberculosis, cancer patients); patients with pacemakers; pregnant females; patients with a positive history of antibiotic use within the past week; teeth with acute pain, mobility degree > II, severe periodontal disease, severely curved or calcified root canals; teeth with internal or external root resorption, vertical or horizontal fractures extending below the cementoenamel junction (CEJ); teeth in which drainage from the canal could not be controlled after access opening, and patients unable to return to the office [19, 20].

All patients were evaluated and recorded by a single clinician including gender, age, tooth location, palpation, periodontal health, percussion tenderness, and fistula on the periapical mucosa and so on. The periodontal condition determination was based on the following clinical parameters [21, 22]: probing depth (PD, the distance from the edge of the gingival margin to the bottom of the gingival sulcus, measured in millimeters), bleeding on probing (BOP, present or absent until 30 s after probing), and clinical attachment level (CAL, the distance from the cementoenamel junction to the bottom of the pocket measured in millimeters, corresponding to the sum of the GR and PD). A periodontal probe (PCPUNC15BR; HuFriedy, Chicago, IL) was used to measure the PD, BOP and CAL in the following 6 sites for each tooth: mesiobuccal, medial, mesiolingual, distolingual, lingual, and distal vestibular. Alveolar bone loss (ABL) assessment was performed by means of periapical X-rays using the paralleling technique. ABL was considered when the distance between the cementoenamel junction and the alveolar bone crest was > 2 mm. The periodontal condition was classified as Table 1 [23].

**Table 1:** Classification criteria of periodontal condition

	PD	CAL	ABL	BOP
Normal	≤ 3 mm	<3 mm	0	(-)
Gingivits	>3 mm	<3 mm	0	(+)
Mild Periodontitis	≤ 4 mm	<3 mm	≤ 7 mm without reaching the apex	(+)
Moderate Periodontitis	>4 ~ ≤ 6 mm	≥ 3~<5 mm	>7 mm without reaching the apex	(+)
Severe Periodontitis	>6 mm	≥ 5 mm	>7 mm reaching the apex	(+)

Abbreviations: PD, probing depth. CAL, clinical attachment level. ABL, Alveolar bone loss. BOP, bleeding on probing

Preoperative radiographs of the roots were each assigned a Periapical Index (PAI) score [24, 25] by 2 blinded, independent, and calibrated examiners as follows:

PAI 1: Normal periapical structure.

PAI 2: Bone structural changes indicating but not pathognomonic for apical periodontitis.

PAI 3: Bone structural changes with some mineral loss characteristic for apical periodontitis.

PAI 4: Well-defined apical radiolucency.

PAI 5: Radiolucency with radiating expansion of bone structural changes.

Multirooted teeth were assigned the highest score for any of the roots. Any disagreement regarding radiographic and clinical examination was resolved by a discussion until final consensus was reached. Examples of PAI 5 category are shown in Fig. 2.

### Randomization

**Fig. 1:**
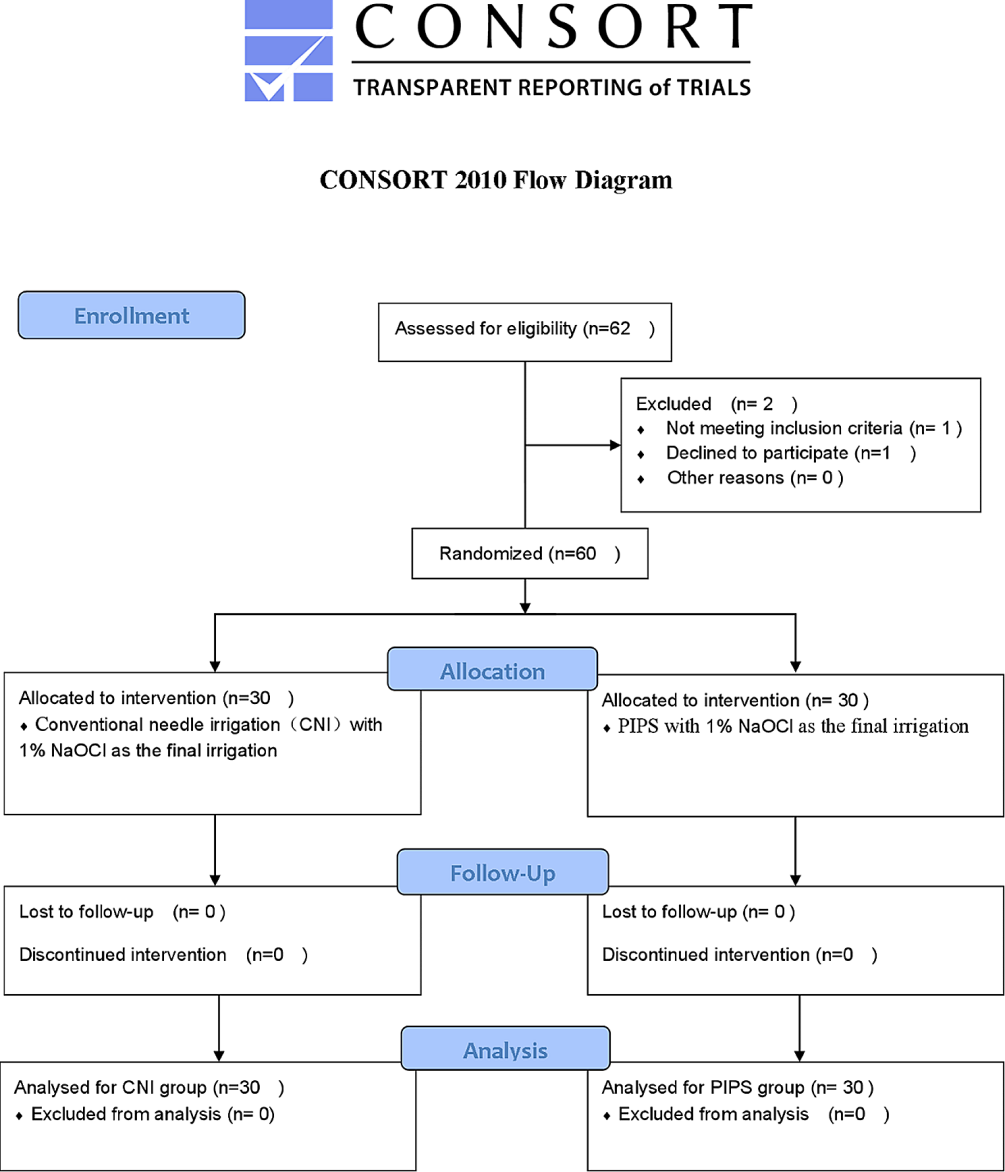
Randomized clinical trial patient flow diagram. Abbreviations: CNI, conventional needle irrigation. PIPS, photon-induced photoacoustic streaming

Randomization was performed using a computer-based program (www.random.org) and a simple random sampling method. The randomization process was carried out by a researcher not involved in the study. Numbers were placed in opaque envelopes and concealed. The envelopes were opened only when the irrigation solution was to be activated. The patients were informed about the study without specifying the group to which they were assigned. The operator determined the activation method for irrigation during the irrigation activation phase. The clinical study commenced after obtaining informed consent from the patients [16, 26]. A total of 60 consecutive adults were included in the study and randomly divided into two groups: the CNI group and the PIPS group (Fig. 1).

**Fig. 2:**
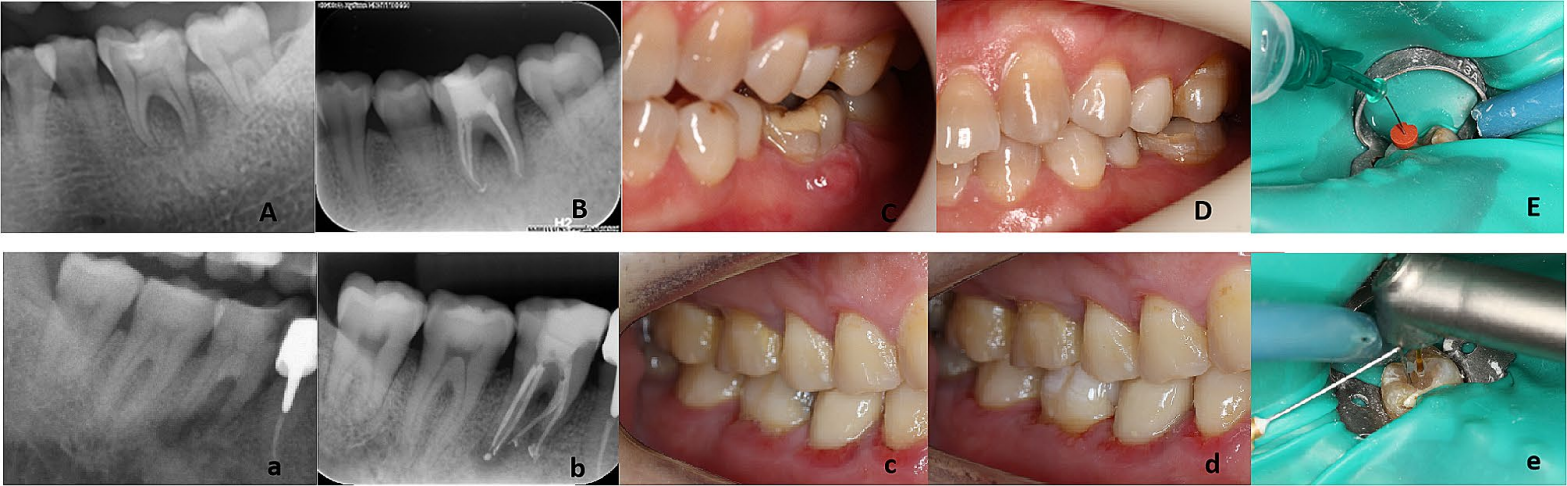
Representative radiographs and intraoral photographs of the treated teeth. A-E from CNI group, a 26-year-old female, complained of gum abscess of the left mandibular first molar for 2 weeks. Preoperative radiographs (A) assigned a PAI 5 score of tooth36#. Postoperative radiographs after root canal obturation (B). Preoperative intraoral photographs (C) showed the presence of fistula, with percussion tenderness, but without periodontitis. Intraoral photographs (D) of 1-week follow-up after root canal preparation showed the absence of fistula. Intraoral photographs (E) showed the final irrigation in CNI group. a-e from PIPS group, a 48-year-old male, complained of rotted teeth in his right mandibular posterior tooth for 1 year. Preoperative radiographs (a) assigned a PAI 5 score of tooth46#. Postoperative radiographs after root canal obturation (b). Preoperative intraoral photographs (c) showed the presence of mesio-proximal caries, with gingivitis, without percussion tenderness and fistula. Intraoral photographs (d) of 1-week follow-up after root canal preparation. Intraoral photographs (e) showed the final irrigation in PIPS group. Abbreviations: CNI, conventional needle irrigation. PIPS, photon-induced photoacoustic streaming. PAI, periapical index

### Root canal treatment

To minimize the risk of cross-infection, the experiment adhered to rigorous aseptic procedures. The experimental environment and surrounding objects were maintained as sterile barriers and subjected to thorough disinfection. Rubber dam isolation was applied, and the dental surface was disinfected using a 30% hydrogen peroxide solution. The root canal treatment was carried out within the confines of the rubber barrier. Strict ATP testing was conducted in the laboratory subsequent to the root canal sampling.

The endodontic treatment was performed by an experienced clinician whose endodontic technique had been calibrated for consistency. All treatments were carried out by this clinician to minimize interpersonal variability. The root canal treatment procedure was the same for both groups, except for the final irrigation protocol. In the first visit, rubber dam isolation was placed, and endodontic access was obtained using a high-speed handpiece and carbide burs. After caries and/or restorations were removed, when necessary, a four-wall structure was rebuilt to provide an irrigation reservoir and support the temporary restoration. The canal was scouted with a size 10-K file (Dentsply Sirona, Ballaigues, Switzerland), and the working length was established using an electronic apex locator (PropexPixi; Dentsply Sirona). The root canal was prepared using MTWO rotary files (VDW, Munich, Germany) and an electric motor (VDW). The following files were used: #10/0.04, #15/0.05, #20/0.06, and #25/0.06. The root canal instrument was used in a single-length technique, with a gentle in-and-out motion until the working length was reached. After each file was used, approximately 3 mL of saline was used for canal irrigation.

Following the preparation, sterile paper points (Dayading, Tianjing, China) were inserted into the root canals to collect bacteria (referred to as Sample A). Subsequently, the 60 patients were randomly divided into two groups based on the assigned final irrigation protocol (detailed information was presented in *2.6 Final Irrigation*). In the CNI group (*n* = 30), the root canals were irrigated with 1% NaOCl + CNI. In the PIPS group (*n* = 30), the root canals were irrigated with 1% NaOCl + PIPS.

After the final irrigation, each root canal was irrigated with 1 mL of saline to remove any residual irrigation solution. Sterile paper points were then inserted into the root canals to collect bacteria (referred to as Sample B). The canals were dried using paper points and sealed with calcium hydroxide paste (Longli, Wuhan, China). The access opening was temporarily filled with a Cavit restoration (ESPE dental, Seefeld, Germany).

All patients were scheduled for a follow-up appointment after 7 days to proceed with canal filling. They were provided with postoperative instructions and given an optional prescription for analgesics. During the second appointment, the condition of the teeth was recorded when the patients returned after 7 days. Teeth showing no signs or symptoms and absence of exudate were considered ready for canal filling. The root canals were irrigated, dried, and filled with gutta-percha (Autofit GuttaPercha points; SybronEndo, Glendora, CA, USA) and an epoxy resin sealer (AH Plus; Dentsply DeTrey GmbH, Konstanz, Germany) using the continuous wave of compaction technique.

### Final irrigation

In the first visit, following the root canal preparation, the 60 patients were randomly divided into two groups based on the assigned final irrigation protocol.

In the CNI group (Fig. 2E), a 30-gauge side needle tip (Endo-Eze; Ultradent Products Inc., South Jordan, UT, USA) was inserted to a depth of 1 mm short of the working length of the root canal system [27]. Each root canal was washed for 30 s with 3 mL of 1% NaOCl.

In the PIPS group (Fig. 2e), the tip was positioned stationary in the pulp chamber and activated, ensuring that the canal and pulp chamber remained passively filled with the irrigation solution throughout the process. The irrigation was performed using a 2,940 nm Er:YAG laser (AT Fidelis; Fotona, Ljubljana, Slovenia) equipped with a handpiece (R14-PIPS, Fotona) holding a 400-µm-diameter quartz tip (XPulse 400/14, Fotona). The tip was applied with the recommended settings of 0.3 W, 15 Hz, and 20 mJ per pulse, without water/air spray. The irrigation in the root canals was activated for 30 s with 3 mL of 1% NaOCl.

### Bacterial sampling

Sample A was collected before the final irrigation, while Sample B was collected after the final irrigation, followed by irrigation of each root canal with 1 mL of saline to remove any residual irrigation solution. Sterile cotton balls were used to remove saline from the access cavity. Subsequently, sterile paper points were inserted into the root canal and left in place for 1 min to collect bacteria. Samples from each canal were placed in separate Eppendorf tubes. Finally, the Adenosine 5’-triphosphate (ATP) levels of bacteria in the samples were determined by measuring luminescence intensity using an ATP fluorescence detector (Lux-T020, Biolight Biotechnology, Guangzhou, China).

### ATP assay kit analysis

ATP in the root canal system was quantified following a previously reported method [28], utilizing an ATP assay kit (S0026, Beyotime, Shanghai, China) according to the manufacturer’s instructions. Each sample of bacteria was collected by sequentially placing sterile paper points in the root canal after irrigation. The paper point was inserted into the root canal and left in place for 1 min to obtain a bacterial sample. The paper point was then transferred to a 1.5 mL Eppendorf tube containing 200 µL of lysis buffer with 0.025 g of glass beads (D3350-01, Omega Biotek Inc, Norcross, Georgia, USA). The tube was centrifuged at 12,000 r/min for 5 min at 4 °C to collect the supernatant. Subsequently, the ATP detection solution was prepared by mixing 20 µL of ATP detection solution with 80 µL of diluted solution in a detection tube. The mixture was placed at room temperature for 5 min. Finally, 20 µL of the bacterial sample was added to the ATP detection solution, and the ATP content was quantified using an ATP fluorescence detector.

### Statistical analysis

Statistical analysis was performed using IBM SPSS R version-4.3.0 (IBM, Armonk, NY). The data obtained were analyzed descriptively and inferentially. The randomization, group assignment, and statistical analysis were performed by a blinded operator. Categorical variables were analyzed using the Pearson Chi-square test or two sample t-test. The distribution of ATP value data was evaluated using the Kolmogorov–Smirnov normality test. The correlation between the demographic data, clinical or radiographic findings and the incidence of pre-operative percussion tenderness or fistula was analysed by Pearson Chi-square test or Mann Whitney U test. Differences in the PIPS group that exhibited normal distribution were analyzed using the t-test. For other ATP value data that did not exhibit normal distribution, nonparametric tests including the Wilcoxon Signed Ranks Test, Mann-Whitney U, and Kruskal-Wallis Test were used. Furthermore, multivariate linear regression model was used to identify the influence factor on the root canal irrigation. Statistical differences were considered significant at a *P*-value of less than 0.05.

## Results

The demographic data, clinical and radiographic findings of the CNI and PIPS groups were summarized in Table 2. There were no significant differences between the two groups in terms of gender (*P* = 0.793), age (*P* = 0.056), tooth type (*P* = 1), periodontal health (*P* = 0.791), periapical index (*P* = 0.935), pre-operative percussion tenderness (*P* = 1.0), and fistula (*P* = 0.791).

**Table 2:** Demographic data, clinical and radiographic findings

Variable	CNI (*n* = 30)	PIPS (*n* = 30)	Statistical test	*P* value
**Gender**, n (%)			Pearson Chi-square	0.793
Male	17 (56.7%)	18 (60%)		
Female	13 (43.3%)	12 (40%)		
**Age** (y)			Two sample t-test	0.056
Mean ± SD	43.13 ± 11.55	37.8 ± 9.53		
Range	26–67	22–56		
**Tooth type**			Pearson Chi-square	1
Non-molar	13	14		
Molar **Dental arch**	17	16		
Maxilla	18	19	Pearson Chi-square	1
Mandible	12	11		
**Percussion**			Pearson Chi-square	1
Tenderness	15	15		
No tenderness	15	15		
**Palpation**			Pearson Chi-square	0.770
Tenderness	9	7		
No tenderness	21	23		
**Tooth mobility**			Adjusted Pearson Chi-square	1
<I°	26	27		
I-II°	4	3		
**Swelling**			Adjusted Pearson Chi-square	1
Yes	3	3		
No	27	27		
**Periodontal health**			Pearson Chi-square	0.791
Normal	15	15		
Mild Periodontitis	6	5		
Gingivitis	9	10		
**Fistula**			Pearson Chi-square	0.791
Yes	11	12		
No	19	18		
**Periapical index (PAI)**			Pearson Chi-square	0.935
PAI 1–2	6	5		
PAI 3	13	13		
PAI 4–5	11	12		

Abbreviations: CNI, conventional needle irrigation; PIPS, Photon-induced photoacoustic streaming

Group A, Standard endodontic manual irrigation; Group B, PIPS laser-activated irrigation

There were no associations between age, gender, tooth type or pre-operative swelling and pre-operative percussion tenderness or fistula (*P* > 0.05) (Table 3). Similarly, there were no associations between pre-operative percussion tenderness and fistula (*P* = 0.118). Meanwhile, pre-operative palpation, periodontal health and periapical index showed a significant association with pre-operative percussion tenderness, as well as pre-operative fistula (*P* < 0.05) (Table 3). Moreover, no significant differences were identified in ATP values before final irrigation between the PIPS and CNI groups (*P* = 0.280). A strong association was observed between ATP values before final irrigation and pre-operative percussion tenderness (*P* = 0.001) or fistula (*P* < 0.001) (Table 3).

**Table 3:** The correlation between the demographic data, clinical or radiographic findings and the incidence of pre-operative percussion tenderness or fistula

Variable	Statistical test	Pre-operative percussion tenderness	Pre-operative fistula
Gender	Pearson Chi-square	0.190	0.164
Age	Pearson Chi-square	0.096	0.135
Tooth type	Pearson Chi-square	0.836	0.6001
Pre-operative fistula	Pearson Chi-square	0.118	/
Dental arch	Pearson Chi-square	0.026	0.194
Palpation	Pearson Chi-square	0.009	0.009
Tooth mobility	Pearson Chi-square	0.108	0.20
Swelling	Pearson Chi-square	0.197	0.051
Periodontal health	Pearson Chi-square	0.024	0.002
Periapical index (PAI)	Pearson Chi-square	0.012	<0.001
ATP value before final irrigation (Sample A)	Mann Whitney U	0.001	<0.001

A multivariate linear regression model was used to identify the influence factor on the ATP value after final irrigation, which revealed that pre-operative percussion tenderness (*P* = 0.006) and fistula (*P* < 0.001) significantly affected the ATP value after final irrigation. Moreover, the method used in the final irrigation (*P* < 0.001) also significantly affected the ATP value after final irrigation (Table 4).

**Table 4:** A multivariate linear regression model identifying influence factors of ATP value after final irrigation in the root canal

	Regression Coefficient	Std. Error	t	*P* value
(Intercept)	1206.9	131.4	9.182	1.28e-12
Dental arch	225.8	135.2	1.670	0.101
Tooth mobility	402.4	209.4	1.922	0.060
Pre-operative percussion tenderness	418.1	146.6	2.853	0.006
Pre-operative fistula	531.3	151.7	3.502	< 0.001
Final irrigaiton	-978.1	122.2	-8.001	< 0.001

**Fig. 3:**
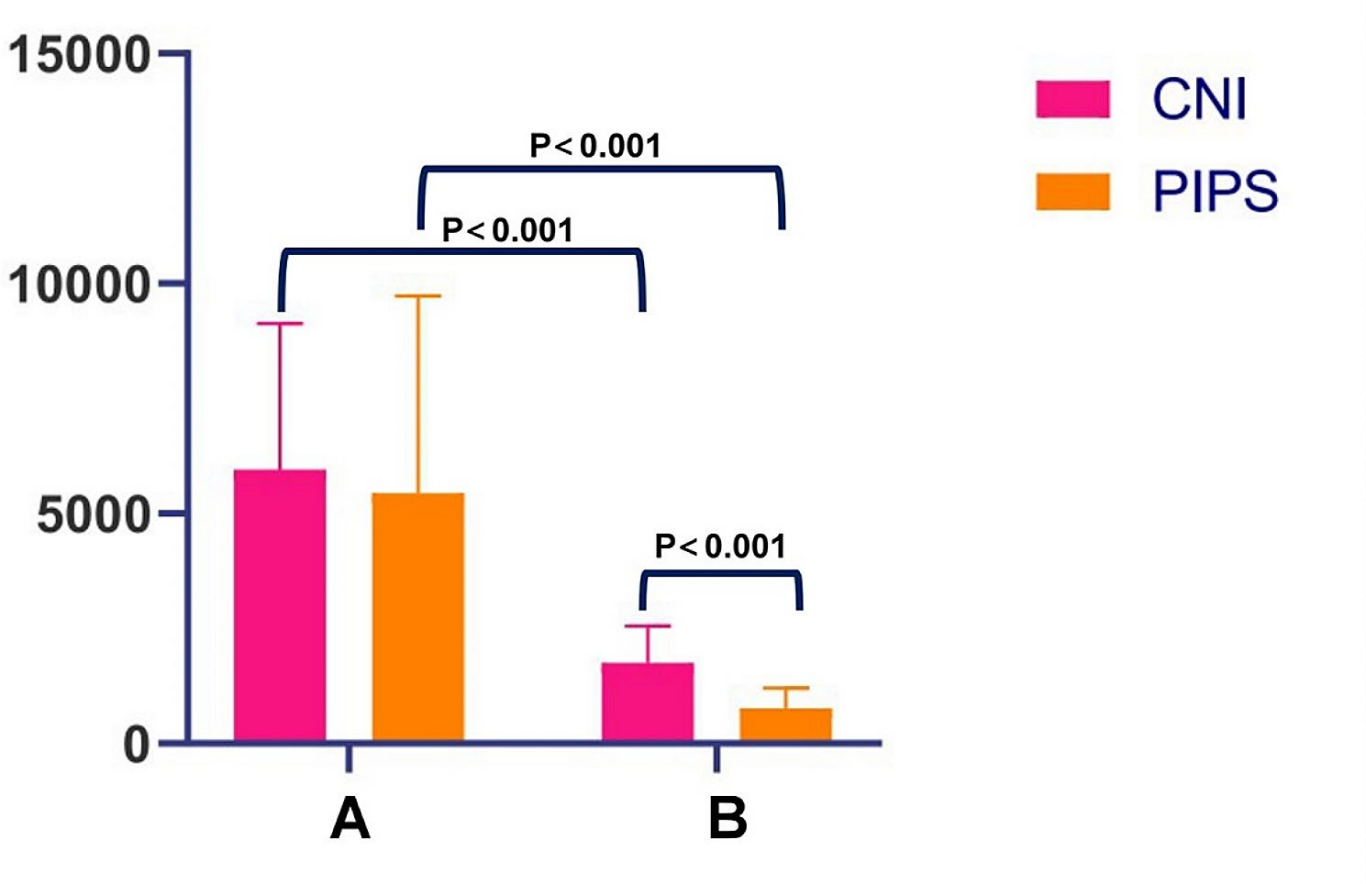
Comparison of ATP values in CNI group and PIPS group. A: ATP value before final irrigation (A); B: ATP value after final irrigation (B). Abbreviations: CNI, conventional needle irrigation. PIPS, photon-induced photoacoustic streaming

Figure 3 illustrated the effect of final irrigation techniques on ATP values. Final irrigation significantly reduced ATP values in both groups (*P* < 0.001). Furthermore, the ATP values after final irrigation was greater in the CNI group compared to the PIPS group (*P* < 0.001).

**Fig. 4:**
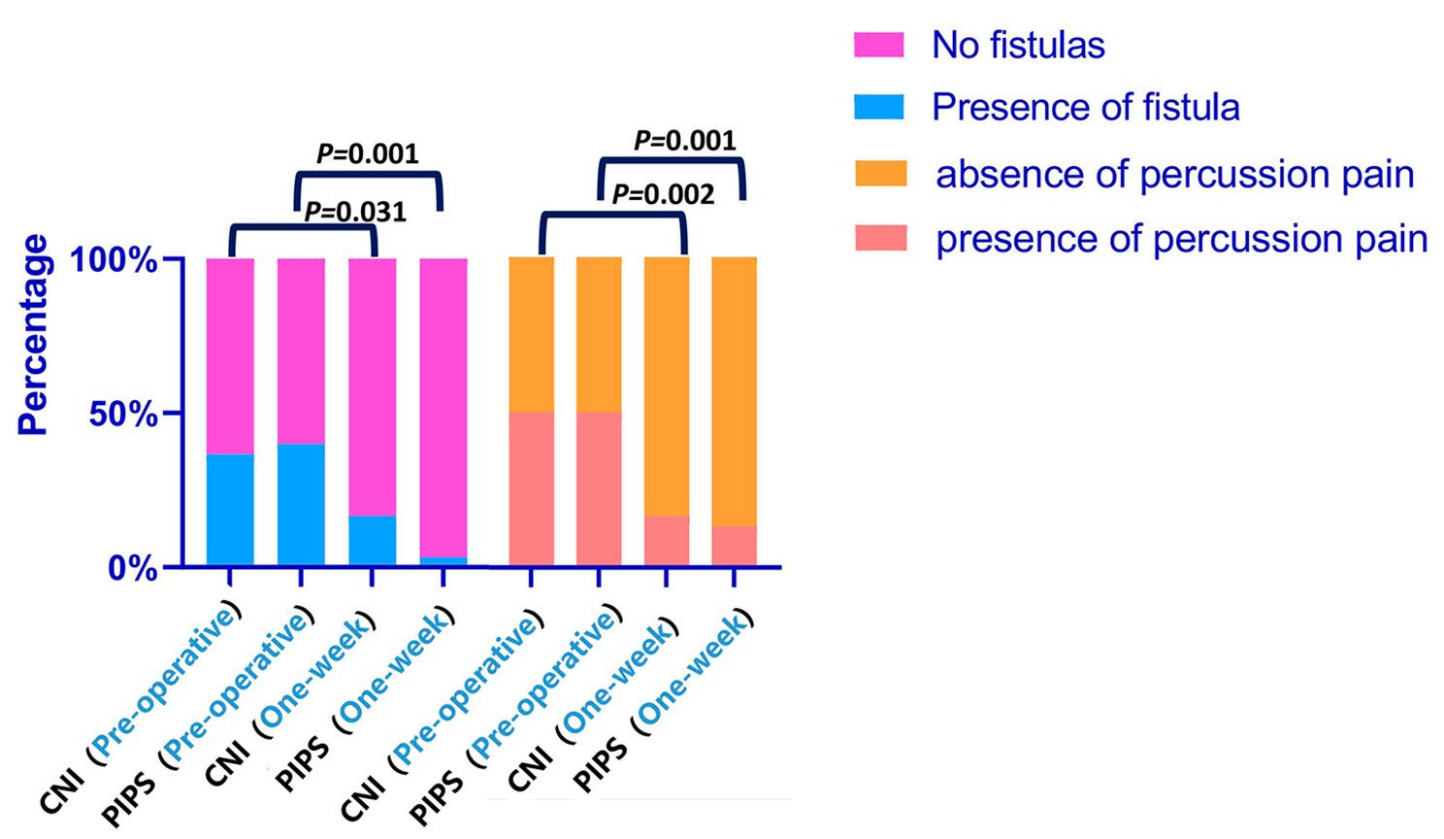
The incidence of percussion tenderness and fistula in the CNI and PIPS groups both pre-operatively and at 7-day follow-up. Abbreviations: CNI, conventional needle irrigation. PIPS, photon-induced photoacoustic streaming

Figure 4 depicted the incidence of percussion tenderness and fistula pre-operatively and at the 7-day follow-up. The incidence of percussion tenderness and fistula was significantly reduced in both the CNI and PIPS groups after 7 days of follow-up (*P* < 0.05). The incidence of fistula and percussion pain was greater in the CNI group than in the PIPS group, although this difference was not statistically significant (*P* > 0.05).

## Discussion

Our clinical results revealed a significant reduction in the ATP value when both PIPS and conventional needle irrigation were used as the final irrigation protocol. Notably, PIPS irrigation exhibited a significantly stronger bactericidal effect than CNI irrigation (Fig. 3). These findings were partially supported by a recent randomized clinical trial [15], which also demonstrated the effectiveness of PIPS irrigation in reducing bacterial counts (measured by colony-forming units) in the root canal system. There is a potential bias in the duration of CNI irrigation, as each root canal was irrigated for 30 s with 3 mL of 1% NaOCl. This duration may slightly differ from the clinical situation where irrigation times could be longer.

It is important to note that although PIPS exhibited disinfectant efficacy in the study conducted by Mandras et al. [15], there was no statistically significant difference compared to CNI for facultative anaerobic and Gram-negative obligate anaerobic strains. The disparities observed between these two studies could potentially be attributed to variations in case selection and the methodology employed for microbiological analysis. Studies have highlighted the limitations of microbial culture assays in accurately quantifying culturable bacteria during biofilm assessment [29, 30]. In contrast, ATP biotechnologies, which rely on nucleic acid measurements, have demonstrated higher sensitivity compared to bacterial culture methods [31]. Among these methods, the quantitative ATP technique has proven to be a convenient and rapid tool for assessing live bacteria through the bioluminescence luciferin-luciferase reaction. This approach has also been successfully employed in detecting infected root canals [32–34]. In addition, in vitro studies have further supported the effectiveness of the PIPS protocol. The study has shown that the PIPS protocol exerts a stronger bactericidal effect in the apical region and effectively removes more microbial biofilm from inside the canal system compared to CNI [13].

The presence of a sinus tract is often indicative of an active infection and can signify the persistence, emergence, or recurrence of apical periodontitis [35]. Additionally, tenderness to percussion is considered a sign of infection and inflammation in root canal-treated teeth [36]. A multivariate linear regression model was employed to determine the factors influencing the ATP value after final irrigation. The analysis revealed that pre-operative percussion tenderness and the presence of a fistula significantly impacted the ATP value after final irrigation, as shown in Table 4. In our study, significant improvements in clinical symptoms, including a decrease in the incidence of percussion pain and fistula, were observed in both the PIPS group and the CNI group (Figs. 2 and 4). The results indicate that root canal disinfection effectively reduces the occurrence of post-operative percussion pain and fistula, as well as lowering the ATP value in the root canal.

Interestingly, PIPS irrigation appeared to have a slightly better impact on the incidence of fistula compared to traditional needle irrigation, although this difference was not statistically significant. While the irrigation method impacted the ATP value in the canal, this effect did not significantly influence clinical symptoms, likely due to the limited sample size. The absence of a control group, which would omit final irrigation, makes it unclear whether the observed reduction in symptoms was attributable to the instrumentation process or the final irrigation itself. Further research with a larger sample and a well-defined control group is needed to clarify this aspect. Several clinical trials have reported that PIPS irrigation results in lower levels and prevalence of post-operative pain when compared to the CNI group [15, 16, 27]. These findings highlight the importance of achieving complete debridement of microbes residing inside the root canal.

## Conclusion

The combination of PIPS and 1% NaOCl irrigation demonstrated a superior disinfection effect compared to CNI, potentially leading to significant enhancements in clinical outcomes. Based on the limitations of our present study, the use of PIPS can be considered a viable modality in the endodontic treatment of teeth with apical periodontitis. These findings provide a foundation for further research with a larger sample size and long-term follow-up, which would allow for a more comprehensive understanding the role of irrigant activation in endodontic treatment.

## Data Availability

The datasets used and/or analysed during the current study available from the corresponding author on reasonable request.
